# Consistent and flexible maternal effects: how the environments of a mother influence the offspring phenotype

**DOI:** 10.1111/brv.70062

**Published:** 2025-08-04

**Authors:** Sin‐Yeon Kim, Judith Morales

**Affiliations:** ^1^ Grupo Ecoloxía Animal, Torre CACTI, Centro de Investigación Mariña, Universidade de Vigo Vigo 36310 Spain; ^2^ Department of Evolutionary Ecology National Museum of Natural Sciences‐Spanish National Research Council (MNCN‐CSIC) c/José Gutiérrez Abascal 2 Madrid 28006 Spain

**Keywords:** animal model, developmental plasticity, life‐history evolution, maternal effects, ontogeny, intergenerational plasticity, quantitative genetics, reaction norm

## Abstract

The environment experienced by a mother influences offspring phenotype through maternal effects, which can have significant adaptive benefits for both the mother and the offspring. However, the ways in which maternal environments influence offspring development are extremely diverse, and empirical studies using an outcome‐based approach often fail to support different maternal effect hypotheses. We argue that this is in part because such studies overlook the ontogeny of the maternal phenotype. Here, we review how the environments experienced by a mother across different life stages influence the development of the maternal phenotype. Then, we propose a new framework that differentiates between two main processes of maternal effects according to the life stage at which a specific maternal trait is developed and how long its effect persists during the mother's reproductive life. The “consistent” maternal phenotype is developed mainly during a mother's early life and consistently affects the phenotype of all offspring produced during her lifetime, whereas the “flexible” maternal phenotype changes in response to environmental conditions experienced during her adult life and affects the phenotype of her subsequent offspring. We review how consistent and flexible maternal effects can contribute to different maternal effect processes, such as condition‐transfer effects, cascading effects, intergenerational plasticity and developmental programming. We also provide empiricists with a quantitative genetic method, which integrates the ontogenetic scope into maternal effect testing, to determine how the early or late environments shape the maternal phenotype across ontogeny and then examine how this maternal phenotype affects offspring phenotype. We highlight that this conceptual and methodological framework of disassembling the multiple processes by which genes and environments interactively influence the maternal and offspring phenotypes will help us to explain the astonishing variation in maternal strategies and life‐history trade‐off patterns.

## I. INTRODUCTION

### (1) Outcome‐based approaches to adaptive maternal effects

**Fig. 1:**
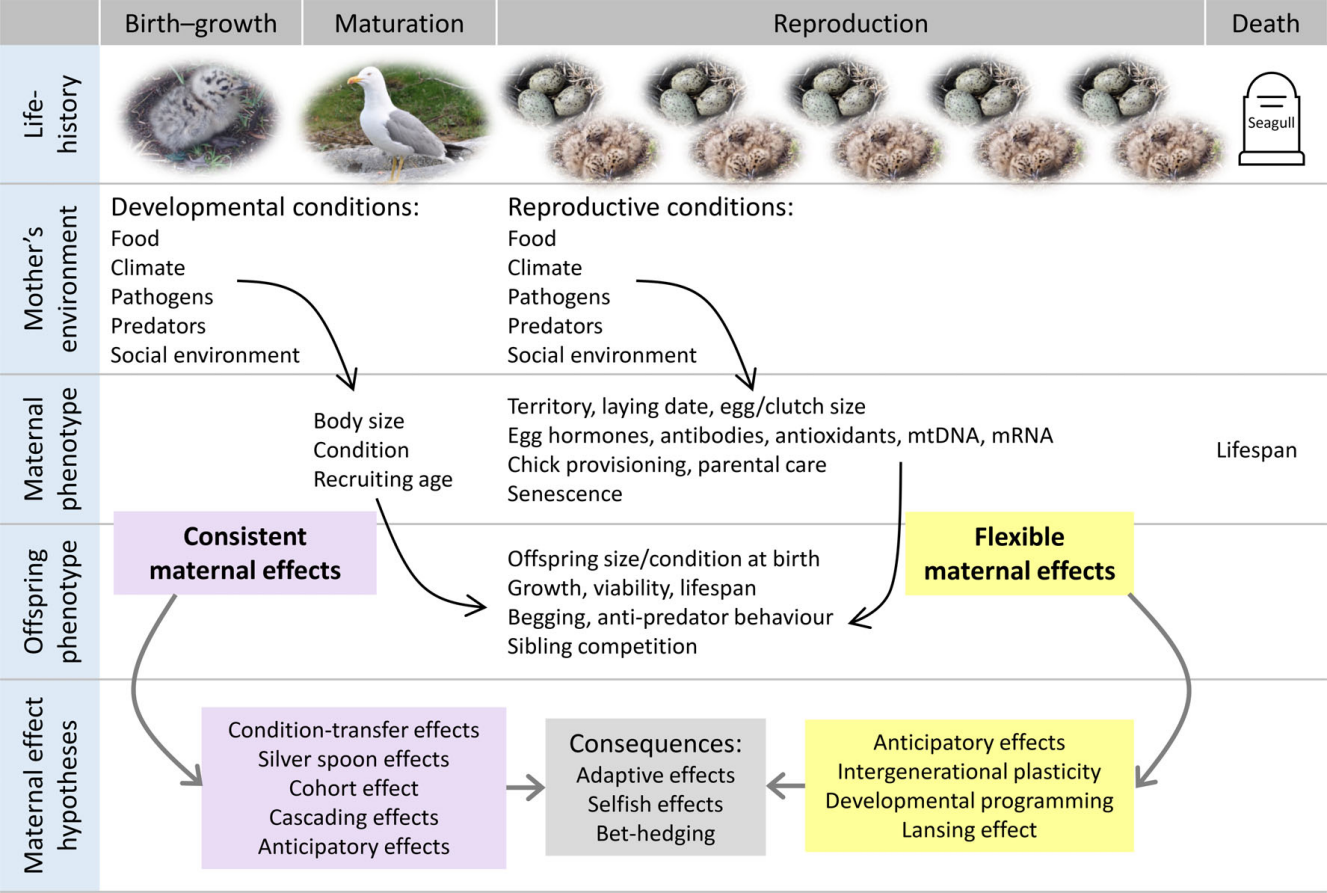
Consistent and flexible maternal effect processes and related hypotheses. Environmental conditions experienced by the mother during her developmental and reproductive phases mainly determine the consistent and flexible maternal phenotypes, respectively, which in turn influences offspring phenotype and fitness through different processes. mRNA, messenger RNA; mtDNA, mitochondrial DNA.

Maternal effect is defined as any causal influence of the mother's phenotype on her offspring's phenotype either before or after birth (Mousseau & Fox, 1998a, 1998b; Wolf & Wade, 2009). The study of maternal effects has a long history (Mousseau *et al*., 2009), and its major interest and focus has been whether maternal effects are adaptive or not for the mother and/or offspring (Galloway & Etterson, 2007). Thus, Marshall & Uller (2007) suggested the classification of maternal effects according to their consequences, for example, whether the effects (*i*) increase maternal fitness by increasing offspring fitness (i.e. adaptive maternal effects), (*ii*) increase maternal fitness at the expense of offspring fitness (i.e. selfish maternal effects), (*iii*) reduce both maternal and offspring fitness (i.e. transmissive maternal effects), or (*iv*) reduce variance in maternal fitness by producing offspring with a range of phenotypes (i.e. bet‐hedging) (Table 1; Fig. 1). Within this prevailing framework, maternal effects are usually tested using an outcome‐based approach, that is, by studying the fitness consequences of maternal or offspring traits.

**Table 1:** Different forms of consistent and flexible maternal effects and their definitions.

Maternal effects	Definition	References
*Classification according to the consequences of maternal effects*		
Adaptive maternal effects	Maternal effects that increase offspring fitness	Marshall & Uller (2007)
Selfish maternal effects	Maternal effects that increase maternal fitness at the expense of offspring fitness	Marshall & Uller (2007)
Transmissive maternal effects	Maternal effects that reduce both maternal and offspring fitness	Marshall & Uller (2007)
Bet‐hedging	Maternal manipulation of the variance in offspring phenotype in unpredictable environments to ensure that some will survive	Crean & Marshall (2009)
*Classification according to the forms and causes of maternal effects*		
Condition‐transfer effect or fitness inheritance	Transfer of maternal condition to offspring, mediated by maternal investment of nutrients or other substances, affecting both maternal and offspring fitness	Bonduriansky & Crean (2018); Qvarnström & Price (2001)
Silver spoon effect	Life‐long fitness advantage in individual females that develop under favourable early conditions	Grafen (1988); Lindström (1999)
Cohort effect	Fitness variation among cohorts due to variation in early developmental conditions	Lindström & Kokko (2002)
Cascading effect	Maternal effects that influence the same maternal traits of offspring	McGlothlin & Galloway (2014); Pick *et al*. (2019)
Anticipatory effect or predictive adaptive responses	Maternal alteration of offspring phenotype according to the anticipated post‐natal environment	Agrawal *et al*. (1999); Fox *et al*. (1997)
Intergenerational plasticity	Plasticity of offspring phenotype in response to the environment experienced by the mother	Bonduriansky (2021); Mousseau & Fox (1998b)
Developmental programming	Developmental plasticity that alters future phenotypes in response to the developmental environment	West‐Eberhard (2003, 2005)
Lansing effect	Negative maternal age effects on the lifespan of offspring	Lansing (1947); Monaghan *et al*. (2020)

From the offspring's perspective, the maternal phenotype is a cue for environmental conditions, and selection may favour offspring plasticity to this cue (Uller, 2008; Wells, 2007). From the mother's perspective, on the other hand, selection may shape the maternal response to the environment to optimise the cue transmitted to the offspring. Adaptive offspring plasticity mediated by maternal and paternal effects has been described in different hypotheses (Fig. 1; Table 1), including among others anticipatory effects (Agrawal, Laforsch & Tollrian, 1999; Fox, Thakar & Mousseau, 1997; Marshall & Uller, 2007), adaptive intergenerational plasticity (Bonduriansky, 2021; Galloway & Etterson, 2007; Mousseau & Fox, 1998b) and adaptive developmental programming (Horton & Stetson, 1990; West‐Eberhard, 2003). Despite subtle differences in their focus and context, all these hypotheses explain different parental effect processes that induce adaptive offspring plasticity (Table 1; Badyaev & Uller, 2009; Bonduriansky & Crean, 2018; Crean & Bonduriansky, 2014; Marshall & Uller, 2007). Importantly, these hypotheses predict that such parental strategies will maximise offspring fitness when the parents express an appropriate cue, and the parental and offspring environments are indeed matched (Marshall & Uller, 2007; Mousseau & Fox, 1998a). It is interesting to note, however, that a meta‐analysis of experimental studies with a fully factorial design showed only weak evidence for higher offspring performance when parental and offspring environments are matched, while the principal determinant was the postnatal environment (Uller, Nakagawa & English, 2013).

One of the potential reasons for the failure to establish the strong adaptive nature of intergenerational plasticity and developmental programming may be that most empirical studies focus only on offspring plasticity and overlook the ontogeny of the maternal phenotype, as previously acknowledged (Uller, 2008; Uller *et al*., 2013). While many studies of maternal effects use some general estimate of maternal effects, such as egg size or offspring provisioning, it is typically poorly understood how the maternal environment affects the maternal phenotype across different life stages. This lack of an ontogenetic perspective may lead to erroneous predictions. For example, in poor environments, mothers may allocate more resources to the developing embryo (at the cost of reduced fecundity) to compensate for unfavourable conditions that it will encounter after birth. However, within the framework of the previous hypotheses, we would consider this maternal compensatory strategy an inaccurate cue for the offspring regarding the postnatal environment (Sultan, 1996; Uller & Pen, 2011). Thus, it is possible that previous studies could not distinguish between anticipatory and compensatory maternal effects (Badyaev, 2008; Radersma *et al*., 2018). Moreover, in some systems, persistent fitness costs derived from poor maternal environments may outweigh any positive maternal effect that could buffer against or compensate for the environmental conditions, thereby limiting the opportunity to detect adaptive maternal effects and the benefits of environmental matching.

### (2) Towards understanding maternal effect mechanisms along the ontogenetic continuum

To understand fully the evolution of maternal effects and its consequences, it is necessary to disassemble complex maternal effect processes, and first to determine how the involved maternal traits develop across the ontogenetic continuum (Räsänen & Kruuk, 2007; Wolf *et al*., 1998). Although the phenotype is most responsive to inputs from the environment and the genome during early development, individual responses to changing environmental conditions can induce phenotypic change also during adulthood (Piersma & Drent, 2003; West‐Eberhard, 2003). Thus, whether the environment induces changes in a mother's phenotype during her early development or her later life (especially during the reproductive stage) is a relevant distinction in terms of the potential consequences for the mother and the offspring. For example, different food supplementation experiments showed that reproductive rate and offspring size are influenced only by the mother's juvenile environmental conditions, irrespective of adult environmental conditions, in the cichlid fish *Simochromis pleurospilus* (Taborsky, 2006b), whereas the adult environment has an overriding effect on the reproductive success of females in the bank vole (*Myodes glareolus*) (Helle, Koskela & Mappes, 2012).

The developmental timing of a specific maternal phenotype across the ontogenetic continuum should influence how long its effect persists. Maternal traits developed mainly during the mother's early life should be related to consistent differences among females in individual quality (Fox & Savalli, 1998), within the axis of phenotypic variation related to individual fitness (Wilson & Nussey, 2010). By contrast, some other maternal traits may form mostly during the mother's reproductive life by genes expressed at this stage and change flexibly within individuals in response to social and other environmental conditions experienced during each reproductive event (Hernández *et al*., 2020; Räsänen & Kruuk, 2007; Strickland *et al*., 2021). Although within‐individual consistency and flexibility are two important attributes of maternal effects, and can have different ecological and evolutionary causes and consequences, their conceptual and functional differences have rarely been discussed.

While the outcome‐based approach has been very useful for the study of maternal effects as adaptations, the ontogenetic view of the maternal phenotype shifts the focus onto the mechanisms underlying different maternal effect processes. Such mechanisms of maternal effects can be revealed by studying how the early developmental or reproductive environment of a female shapes her phenotype, which includes morphological, physiological, behavioural and life‐history traits. For example, resource availability and competition during early life influences maternal size and condition at maturation (Douhard *et al*., 2013; Hopwood, Moore & Royle, 2014; Pfennig & Martin, 2009), and diverse external stimuli during reproduction, such as pathogens, mates, predators, food and climate, affect the transfer of maternal antibodies (Boulinier & Staszewski, 2008; Gauzere *et al*., 2021), nutrients (Osorno *et al*., 2006), hormones (Crocker & Hunter, 2018; Giesing *et al*., 2011; Groothuis *et al*., 2019) and antioxidants (Morales, 2020; Royle, Surai & Hartley, 2001) to the offspring. The intensity of maternal care received when young influences care‐giving behaviour of adult rats (Champagne *et al*., 2003; Champagne & Meaney, 2007), and parents change their nest‐site selection and breeding phenology in response to current climate factors, such as temperature and humidity (Charmantier *et al*., 2008; Pruett, Fargevieille & Warner, 2020). While these examples represent widely known parental effect drivers, more recent studies have shown that both the developmental and reproductive environments of females can influence the offspring phenotype also through maternal gene products, such as messenger RNA (mRNA), microRNA and mitochondrial DNA (mtDNA) copies, which are transferred to oocytes and support offspring development during early embryogenesis (Kim *et al*., 2022, 2023; Marlow, 2020; McJunkin, 2018; Wei *et al*., 2019). Similarly, paternal obesity at reproduction is known to affect the development and behaviour of the offspring through sperm‐borne epigenetic changes (Bodden, Hannan & Reichelt, 2020; Tomar *et al*., 2024). As the parental phenotype itself has often been overlooked in parental effect studies (Pick, Postma & Tschirren, 2019), understanding its development may allow us to bridge the gap between adaptive parental effect hypotheses and empirical support.

In this review and synthesis, we aim to provide a new framework to support studies on the mechanisms and developmental processes of maternal effects. We do not attempt to replace existing frameworks, which focus on adaptation, because they consider different aspects of the evolution of maternal effects (listed and summarised in Table 1). We instead propose distinguishing between two different attributes of maternal effects, according to the ontogenetic stage at which the maternal trait affecting the offspring's phenotype is developed and how long its effect persists, namely “consistent” and “flexible” maternal effects (Fig. 1). The consistent and flexible maternal phenotypes are mostly determined at different life stages, that is during early developmental and reproductive phases, respectively (Fig. 1). The consistent maternal phenotype will affect all offspring produced during the lifetime, whereas the flexible maternal phenotype will affect mainly the offspring produced in that reproductive season.

In the subsequent sections, we first describe the concepts of consistent and flexible maternal effects, explain how they connect with the main hypotheses of maternal effects, and review the previous approaches taken by empirical studies to explore such hypotheses (Section II). Quantitative genetic methods can be very useful for incorporating the ontogenetic scope into maternal effect studies because they explore how phenotypic variations in maternal traits are determined by genetic and environmental effects. Thus, we introduce how to test both types of maternal effects by using advanced quantitative genetic approaches (Section III). Lastly, we discuss some outstanding open questions, which can be explored best by using our conceptual and methodological framework of consistent and flexible maternal effects (Section IV). The two concepts we explore are relevant to many maternal traits and to all animal species with a variety of life‐history strategies. Indeed, there is scope for either iteroparous or semelparous mothers to adjust maternal transfer in response to their own or their offspring's developmental environments, regardless of whether they lay eggs or give birth to developed young (Houston *et al*., 2006). We suggest that distinguishing the two attributes at both the conceptual and practical level can advance our understanding of the evolution and ecology of maternal effects.

## II. CONSISTENT AND FLEXIBLE MATERNAL EFFECTS

We define a consistent maternal effect as “a maternal effect mediated by the consistent maternal phenotype developed mainly during the mother's early life developmental phase”, and a flexible maternal effect as “a maternal effect mediated by the maternal phenotype that changes in response to the environment experienced during the mother's reproductive life”. To distinguish between consistent and flexible maternal phenotypes, we use concepts from developmental and evolutionary biology and attempt to integrate the two approaches. Development results from the interplay between genes, cellular processes and environmental conditions (Müller, 2007; Waddington 1942; West‐Eberhard, 2003), and early‐life developmental plasticity is an important property of organisms enabling them to produce consistently distinct phenotypes in response to the environment experienced early in life (Parsons *et al*., 2020; Pfennig, 2021; Pigliucci, 2001; West‐Eberhard, 2003). Organisms can also continuously transform their phenotypes beyond the early developmental phase in response to changing environmental conditions during adulthood by phenotypic flexibility (Piersma & Drent, 2003). Recent studies have shown that these environmentally induced phenotypes can provide variation on which selection will act (Lala *et al*., 2024; Levis & Pfennig, 2016, 2020; Parsons *et al*., 2016; Sultan, 2021). Thus, reaction norms of maternal traits across the ontogenetic continuum may have important consequences for the evolution of maternal effects, life histories and offspring responses.

### (1) Developmental environment and consistent maternal effect processes

Given that a disproportionately large part of phenotypic organisation occurs during an early and brief window of development (West‐Eberhard, 2003), the environment experienced by an individual during its early life and the underlying genetics during this period produces persistent effects that last throughout adulthood (Lindström, 1999; Lummaa & Clutton‐Brock, 2002). Like many other traits, maternal traits of an individual female can be determined during this early developmental stage and maintained during her lifetime (Jonsson & Jonsson, 2014). These consistent maternal phenotypes will manifest (with a delay) at maturity and affect the phenotype of all her offspring produced throughout life (Burton & Metcalfe, 2014).

Understanding how the early developmental environment of a female shapes her long‐lasting maternal phenotype is a useful approach by which different hypotheses of maternal effect process can be tested or discussed (see the *Classification according to the forms and causes of maternal effects* in Table 1). For example, the condition‐transfer hypothesis explains that an individual's condition influences its parental investment, such that high‐condition mothers typically provide more resources or care to their offspring, thereby producing high‐condition offspring (Bonduriansky & Crean, 2018; Qvarnström & Price, 2001). The early environments experienced by a mother influence her condition associated with the consistent maternal phenotype (maternal quality), which determines an important part of the developmental environment for her offspring. Thus, the effects of early environmental conditions experienced by the mother and the offspring during their own development can act additively on the offspring phenotype (Engqvist & Reinhold, 2016; Rodríguez‐Ruiz, López & Martín, 2020). An interesting question is whether this condition‐transfer effect is an adaptive maternal effect, and the answer may depend on whether the offspring encounter environments matching or mismatching the mother's experience.

Similarly, consistent maternal effects may play an important role in fitness inheritance through the persistent effects of early developmental conditions, as predicted by the silver spoon or cohort effect hypotheses. Individuals that develop under favourable conditions during early life (silver spoon) or are born in favourable years (cohorts) have a life‐long advantage and consistently outperform those developing under adverse conditions, thereby inducing variation among individuals and cohorts in average fitness (Grafen, 1988; Lindström, 1999; Lindström & Kokko, 2002). Such persistent advantage induced by favourable conditions during early development is likely to appear also in the maternal phenotype of those lucky individuals or cohorts. Thus, silver spoon and cohort effects of developmental environments can be transferred, *via* consistent maternal effects, to subsequent generations (Beckerman *et al*., 2002). For example, in the common lizard (*Lacerta vivipara*), climatic conditions during early development have delayed life‐history effects on adult maternal traits, thereby influencing the offspring phenotype (Marquis, Massot & Le Galliard, 2008; see Table 2).

**Table 2:** Selected examples exploring maternal effect processes determined by the developmental environment of the mother. Developmental environment refers to natural or experimental conditions experienced by the mother during its own development that result in consistent maternal effects.

Species	Maternal traits explored	Offspring traits explored	Developmental environment of the mother	Maternal effect hypothesis	Statistical method	References
Invertebrates						
Rotifers (*Brachionus calyciflorus*)		Life‐history traits; starvation resistance	Food quality manipulated	Selfish maternal effects; adaptive effect; intergenerational plasticity; anticipatory effect	LMM (one neonate per female)	Zhou & Declerck (2020)
Water flea (*Daphnia cucullata*)		Helmet growth; survival	Predation risk manipulated	Adaptive effect; anticipatory effect	LM (family average analysed)	Agrawal *et al*. (1999)
Burying beetle (*Nicrophorus vespilloides*)	Egg size	Body mass	Food availability manipulated	Fitness inheritance; condition transfer effect	LMM (family as a random effect)	Steiger (2013)
House cricket (*Acheta domesticus*)	Ecdysteroid hormones	Growth rate; daughter's fecundity	Diet quality manipulated	Silver spoon effect; fitness inheritance; intergenerational plasticity	LMM (family as a random effect)	Crocker & Hunter (2018)
Fishes						
Three‐spined stickleback (*Gasterosteus aculeatus*)	Clutch size and mass; egg carotenoids		Winter temperature manipulated	Adaptive effect; anticipatory effect	LMM (clutch order within mother as random intercept and slope)	Kim *et al*. (2017)
Three‐spined stickleback (*Gasterosteus aculeatus*)	mtDNA copy number	Body mass; survival	Winter temperature manipulated	Adaptive effect; anticipatory effect	LMM (clutch order nested within mother as random effects)	Kim *et al*. (2022)
Cichlid spp. (*Simochromis pleurospilus*)	Egg mass	Size	Food availability manipulated	Adaptive effect; intergenerational plasticity; anticipatory effect	LM (mother average analysed)	Taborsky (2006a)
Amphibians						
Moor frog (*Rana arvalis*)	Egg size; egg capsule composition	Size; survival	Acid stress in population of origin	Adaptive effect; intergenerational plasticity	LM (mother average for egg size); GLMM (family as a random effect)	Räsänen *et al*. (2003a, 2003b)
Spadefoot toads (*Spea multiplicata*)	Egg size	Morph (large carnivore *vs* small omnivore)	Presence of heterospecific competitor	Adaptive effect	LM (mother average analysed)	Pfennig & Martin (2009)
Reptiles						
Common lizard (*Lacerta vivipara*)	Litter size	Size; survival	Rainfall	Cohort effect; intergenerational plasticity	LMM (mother nested within cohort as random effects)	Marquis *et al*. (2008)
Birds						
Japanese quail (*Coturnix japonica*)	Egg size	Egg size	Selected lines for low *vs* high maternal investment	Cascading effect	AM; LMM (mother as a random effect)	Pick *et al*. (2019)
Zebra finch (*Taeniopygia guttata*)		Adult mass; condition; colouration	Proxies of nutritional status (e.g. laying and hatching order, brood size)	Adaptive effect Condition transfer effect; anticipatory effect; intergenerational plasticity	AM; LMM (group as a random effect)	Pei *et al*. (2020)
Mammals						
Bank vole (*Myodes glareolus*)	Breeding date; litter size	Survival; body mass; physiological parameters	Food availability manipulated	Adaptive effect; anticipatory effect	LMM (mother as a random effect)	Helle *et al*. (2012)
Bank vole (*Myodes glareolus*)		Growth; survival	Food availability and social environment manipulated	Adaptive effect; anticipatory effect; silver spoon effect; intergenerational plasticity	GLMM (litter as a random factor)	Van Cann *et al*. (2019)
Norway rat (*Rattus norvegicus*)	Maternal care (licking, grooming and nursing)	Maternal care (licking, grooming and nursing)	Stress manipulated	Cascading effect; intergenerational plasticity	LM (mother average analysed)	Francis *et al*. (1999)
Red deer (*Cervus elaphus*)	Plasticity in calving date	Survival	Population density	Adaptive effect; intergenerational plasticity	LMM (mother and mother × rainfall as random effects)	Nussey *et al*. (2005a)

Abbreviations: AM, animal model; GLMM, generalised linear mixed‐effect model; LM, general linear model (*t*‐test, ANOVA or ANCOVA); LMM, linear mixed‐effect model; mtDNA, mitochondrial DNA.

On the other hand, cascading maternal effects occur when a specific maternal trait of a mother either positively or negatively affects the same maternal trait of her female offspring *via* non‐genetic effects (McGlothlin & Galloway, 2014). Examples of negative cascading maternal effects may be found in clutch or litter size, which is typically traded off against offspring body size, and this in turn constrains clutch or litter size when the offspring reproduce themselves (Smith & Fretwell, 1974). A study in the Japanese quail (*Coturnix japonica*) demonstrated positive cascading effects of maternal egg size on offspring egg size (Pick *et al*., 2019; Table 2). Similarly, in rats (*Rattus norvegicus*), licking and grooming behaviours of foster mothers have positive cascading effects on the offspring's maternal behaviours (Francis *et al*., 1999; Table 2). These examples show that the patterns of maternal resource investment and care shape the environment that the offspring experience during early development, which can have persistent effects on the same maternal traits in the daughters.

Finally, anticipatory maternal effects alter the offspring phenotype according to the anticipated environment that they will encounter after birth. In short‐lived animals, in particular, mothers and their offspring often develop in similar conditions. Thus, preparing the offspring for the conditions experienced by the mother should provide adaptive benefits. A classic example in *Daphnia cucullata* has shown that offspring born to mothers exposed to predators before sexual maturity grow helmet‐like anti‐predator defences (Agrawal *et al*., 1999; Table 2), and that this effect persisted throughout various reproductive events of the mothers.

### (2) Reproductive environment and flexible maternal effect processes

When environmental conditions change rapidly and over shorter timescales than a lifetime, it may be beneficial for females to transform continuously and reversibly their maternal strategies. Once a female matures and begins to reproduce, her maternal phenotype can change to some extent in response to the current environmental conditions, that is, those experienced during her adult life. This flexibility of the maternal phenotype is expected to affect in turn the phenotype of her subsequent offspring *via* flexible maternal effects (Wells, 2007). Importantly, in this case, the flexible maternal traits form, and the affected offspring traits develop during the same reproductive season within which environmental conditions are relatively consistent (Burgess & Marshall, 2014).

As reviewed in Table 3, flexible maternal effect processes are related to many existing maternal effect hypotheses, especially intergenerational phenotypic plasticity, anticipatory effect and developmental programming, as well as some hypotheses explained above in the perspective of consistent maternal effects (Fig. 1). In fluctuating environments, the intergenerational process of phenotypic plasticity can allow mothers and offspring to perform better in their common environments (Bonduriansky, 2021; Marshall & Uller, 2007). Indeed, this flexible maternal strategy has been recognised as an important mechanism that buffers populations against rapid environmental changes, such as climate change and pollution (Badás, Romero‐Haro & Morales, 2025; Castano‐Sanz, Gomez‐Mestre & Garcia‐Gonzalez, 2022; Donelson *et al*., 2012; Lind *et al*., 2020; Salinas & Munch, 2012; Shama *et al*., 2014).

**Table 3:** A selected set of examples exploring maternal effect processes determined by the reproductive environment of the mother. Reproductive environment refers to conditions experienced by the mother during her adult life that result in flexible maternal effects.

Species	Maternal traits explored	Offspring traits explored	Reproductive environment of the mother	Maternal effect hypothesis	Statistical method	References
Arthropods						
Leaf beetle (*Stator limbatus*)	Egg mass		Host plant type manipulated	Adaptive effect; anticipatory effect; intergenerational plasticity	LM (repeated measures)	Fox *et al*. (1997)
Seed beetle (*Callosobruchus maculatus*)	Longevity; fecundity; LRS	Longevity; fecundity; LRS	Pesticide concentration manipulated	Transgenerational plasticity	LM for F0 and F1 (one individual per cross); LMM for F2 (grandparent as a random effect)	Castano‐Sanz *et al*. (2022)
Fishes						
Southern pygmy perch (*Nannoperca australis*)	Egg size; fecundity; gonad mass		Hydrological indices	Adaptive effect; bet‐hedging; intergenerational plasticity	LMM (mother nested within sample nested within population as random effects)	Morrongiello *et al*. (2012)
Three‐spined stickleback (*Gasterosteus aculeatus*)	Egg mtDNA copy number	Body mass; survival	Clutch order (time of year)	Adaptive effect; anticipatory effect	LMM (clutch order nested within mother as random effects)	Kim *et al*. (2022)
Three‐spined stickleback (*Gasterosteus aculeatus*)	Egg mRNA transcripts	Survival	Age	Lansing effect	Cox proportional hazard model (family and mother as random effects)	Kim *et al*. (2023)
Daffodil cichlid (*Neolamprologus pulcher*)	Egg mass; protein content	Body mass; survival; anti‐predator behaviour	Predation risk manipulated	Adaptive effect; anticipatory effect; intergenerational plasticity	LMM; GLMM (brood as a random effect)	Sharda *et al*. (2021)
Reptiles						
Common lizard (*Lacerta vivipara*)	Litter size	Size; survival	Temperature	Cohort effect; intergenerational plasticity	LMM (mother nested within cohort as random effects)	Marquis *et al*. (2008)
Carpetan rock lizard (*Iberolacerta cyreni*)		Dispersal; locomotor capability	Nutrient availability manipulated	Anticipatory effect; condition transfer effect	GLMM (mother as a random effect)	Rodríguez‐Ruiz *et al*. (2020)
Birds						
Common guillemot (*Uria aalge*)	Laying date		NAO index	Adaptive effect	LMM (mother and mother × NAO as random effects)	Reed *et al*. (2006)
Great tit (*Parus major*)	Yolk thyroid hormone concentration		Temperature; year	Adaptive effect; anticipatory effect; intergenerational plasticity	LMM (mother, clutch and mother/clutch × temperature as random effects)	Hsu *et al*. (2019)
Great tit (*Parus major*)	Laying date		Temperature; food availability; year	Adaptive effect; anticipatory effect	LMM (mother and mother × temperature as random effects); AM	Nussey *et al*. (2005b)
Collared flycatcher (*Ficedula albicollis*)	Laying date		Temperature; rainfall; NAO index	Adaptive effect; cohort effect	LMM (mother and mother × climatic variable as random effects); AM	Brommer *et al*. (2005)
Mammals						
Bank vole (*Myodes glareolus*)	Breeding date; litter size	Survival; body mass; physiological parameters	Food availability manipulated	Adaptive effect; anticipatory effect; intergenerational plasticity	LMM (mother as a random effect)	Helle *et al*. (2012)
Red squirrel (*Tamiasciurus hudsonicus*)	Parturition date		Spring temperature; food abundance	Adaptive effect; cohort effect; intergenerational plasticity	AM; LMM (mother as a random effect)	Reale *et al*. (2003)
Red deer (*Cervus elaphus*)	Calving date		Autumn rainfall	Adaptive effect	LMM (mother and mother × rainfall as random effects)	Nussey *et al*. (2005a)

Abbreviations: AM, animal model; GLMM, generalised linear mixed‐effect model; LM, general linear model; LMM, linear mixed‐effect model; LRS, life‐time reproductive success; mRNA, messenger RNA; mtDNA, mitochondrial DNA; NAO, North Atlantic Oscillation.

The adaptive advantage of flexible maternal effects, compared to consistent maternal effects, is that mothers can adjust their current maternal phenotype to match their offspring's needs more precisely according to the anticipated environmental conditions. Thus, maternal substances or behaviours directed to eggs or embryos may function as cues that anticipate postnatal developmental environments that offspring will encounter (Burton & Metcalfe, 2014) and mediate developmental programming of offspring (Monaghan, 2008) if the environment does not change too rapidly to anticipate. For example, cichlid mothers lay larger eggs with higher protein content when they experience a (manipulated) high‐risk environment, and this results in enhanced anti‐predator behaviour in the following generation (Sharda *et al*., 2021; Table 3). By contrast, the consistent maternal phenotype, which is determined during the female's early life, may be relatively limited in anticipating the developmental environment of the offspring, especially in rapidly changing environments.

The cohort effect can also be considered as a flexible maternal effect process when offspring traits (and not maternal traits) or within‐female variability across years are the focus. Variation in environmental conditions across years shapes flexible maternal investment, which can have long‐lasting consequences for the offspring. In such cases, flexible maternal effects may mediate the cohort effect in the offspring produced across years with different environmental conditions (Marquis *et al*., 2008; Table 3). Thus, it depends on the study question whether the focus is on the consistent or the flexible component of the maternal phenotype (or both). By acknowledging that they are mostly determined by the environments experienced at very different ontogenetic stages, it becomes easier to make accurate predictions about their respective effects and study the underlying mechanisms.

It is important to note that most maternal traits may differ consistently among different individuals, producing consistent effects throughout the lifetime, but also potentially change within reproductively mature individuals in response to environmental conditions, producing flexible effects too. Thus, one cannot unequivocally classify specific maternal traits as either exclusively consistent or flexible, although certain traits are affected to a much larger extent by early developmental conditions than by the adult environment. Consistent and flexible maternal effect processes can rather be distinguished by investigating the degree to which the internal states and/or external environments of females at different life stages influence any specific maternal trait.

### (3) Previous studies in the contexts of consistent and flexible maternal effects

Although the concept of consistent and flexible maternal effects has not been formally defined before, many previous empirical studies have successfully explored either or both maternal effects (see Tables 2 and 3). Here, we review how consistent and flexible maternal effects have been tested in empirical studies, either experimental or non‐experimental, to seek and suggest a more appropriate and efficient way to quantify them (see Section III).

The consistent effects of early‐developed maternal phenotypes have often been studied non‐experimentally, using cohort or population variation (Marquis *et al*., 2008; Räsänen, Laurila & Merilä, 2003b) and assuming that shared environments result in consistent differences in average traits (Lindström & Kokko, 2002). Other studies have also focussed on individual differences in early developmental conditions among females within populations (Pei, Forstmeier & Kempenaers, 2020). Another common approach to detect consistent maternal effects has been directly to manipulate specific conditions in the environment that mothers experience during their own development (Agrawal *et al*., 1999; Bauerfeind & Fischer, 2005; Helle *et al*., 2012; Kim *et al*., 2017, 2022; Steiger, 2013; Taborsky, 2006b; Van Cann *et al*., 2019; Zhou & Declerck, 2020), and to explore the consequences on both maternal and offspring traits.

At the analytic level, the above examples have shown how consistent maternal effects can be detected through comparisons among females exposed to different early environments. Some of those studies used repeated measures of the same individuals across time, in which case the scores have been either averaged in a linear model or included in a mixed‐effect model that controls for mother's identity as a random factor (see Table 2). In such cases, a strong within‐female repeatability and/or among‐female variance may demonstrate the presence of consistent maternal effects (Tschirren *et al*., 2009), although few studies have reported the individual effect or variance (see Table 2). For example, in three‐spined sticklebacks (*Gasterosteus aculeatus*) from an annual population, mothers that experienced experimentally increased temperatures during development consistently and repeatedly laid clutches containing an increased mtDNA content throughout their single reproductive season, as demonstrated by a fixed effect of experimentally manipulated temperature and a random variance explained by maternal identity (Kim *et al*., 2022).

On the other hand, flexible maternal effects have been studied either by experimentally manipulating environmental conditions to which reproductively mature females are exposed (e.g. Castano‐Sanz *et al*., 2022; Rodríguez‐Ruiz *et al*., 2020; Sharda *et al*., 2021), or by using non‐experimental data of environmental parameters and reproduction across different times and/or space (e.g. Hsu *et al*., 2019; Kim *et al*., 2022; Morrongiello *et al*., 2012; Reale *et al*., 2003). In most cases, they used a cross‐sectional rather than a longitudinal approach in which different (unrelated) females were exposed to different conditions (Bauerfeind & Fischer, 2005; Marshall, 2008; Shama *et al*., 2014). This approach can test flexible maternal effects reliably if different groups of females exposed to distinct conditions have similar genetic variation, or if at least this can be assumed by sampling methods. However, flexible maternal effects should ultimately be testable, if possible, as variation within individuals or genetic families along environmental gradients (e.g. variation between clutches of the same female that are laid under varying natural conditions in a longitudinal study; variation between clutches laid by genetically related females that experience different experimental conditions) by using a reaction norm approach (see Section III).

When repeated measures of the same or related individuals are available, typically, flexibility is estimated statistically by means of repeated measures in a linear model (Fox *et al*., 1997) or a mixed model in which female or family identity is fitted as a random intercept (Reale *et al*., 2003) (see Table 3). The presence of a significant effect of the environmental variable on the maternal or offspring trait would indicate that the trend is present within individual females or families and is largely explained by maternal phenotype flexibility. Moreover, if there are longitudinal data across time or environments, the models can be taken one step further by including individual‐based random regression functions (random intercept and slope), which can provide a direct test of individual variation in responsiveness to environmental changes (Table 3). For example, long‐term population studies on the collared flycatcher (*Ficedula albicollis*) and the great tit used an individual mixed modelling approach to examine the reaction norms of egg‐laying date along gradients of spring temperature (Brommer *et al*., 2005; Charmantier *et al*., 2008; Nussey *et al*., 2005b). This approach revealed that individual females were able to track environmental change associated with global warming and adjust their laying date accordingly, and that much of this was due to individual plastic responses, which were either variable among individuals or fixed within the population.

Finally, some previous literature has established quantitative genetic frameworks to explore maternal effects by partitioning additive genetic variance and maternal variance in offspring phenotype (Gauzere *et al*., 2020; Hadfield, 2012; Kruuk, 2004; McAdam, Garant & Wilson, 2014; Räsänen & Kruuk, 2007). The commonly used quantitative genetic approach for testing maternal effects is to include maternal identity as an additional random effect in an animal model or other mixed effect models fitted to an offspring trait (Kruuk, 2004; McAdam *et al*., 2014; Räsänen & Kruuk, 2007; Wilson *et al*., 2010). Indeed, many empirical studies applying this method have successfully demonstrated how much of the phenotypic variation in an offspring trait is determined by non‐genetic maternal effects (e.g. Bonnet *et al*., 2022; Moore, Whiteman & Martin, 2019; Noble *et al*., 2014; Petelle, Dang & Blumstein, 2017; Pick *et al*., 2016). Furthermore, some studies have integrated the reaction norm approach into quantitative genetic models to study how maternal traits change in response to changing environmental conditions (Brommer, Pietiäinen & Kolunen, 2003; Nussey *et al*., 2005a).

However, many previous approaches used to examine maternal effect outcomes have failed to explain how maternal phenotypes develop across environments and to link maternal reaction norms to their consequences. As previously discussed, studying the architecture of maternal phenotypes across ontogeny should improve our understanding of maternal effect processes and mechanisms, and ultimately contribute to resolve the evolution of maternal effects. Thus, in the following section, we propose how to integrate the ontogeny of the maternal phenotype in testing maternal effects, for which quantitative genetics can be a potentially important tool.

## III. A GUIDE TO TEST CONSISTENT AND FLEXIBLE MATERNAL EFFECTS

Perhaps the most appropriate approach to demonstrate intra‐ and intergenerational plasticity of maternal and offspring traits is to perform a common garden experiment where the mothers are exposed to different developmental and/or reproductive environmental conditions (Roff & Wilson, 2014). However, natural variation among different cohorts, habitats and other common environment factors can also provide excellent opportunities for researchers to perform natural experiments testing maternal effect processes. Clonal organisms, pedigree information, or conventional breeding designs (e.g. full‐sib or half‐sib design) may be used in both the common garden and natural common environment approaches if one also aims to test genotype‐by‐environment (G × E) interactions rigorously (Roff, 1997; Roff & Wilson, 2014).

**Fig. 2:**
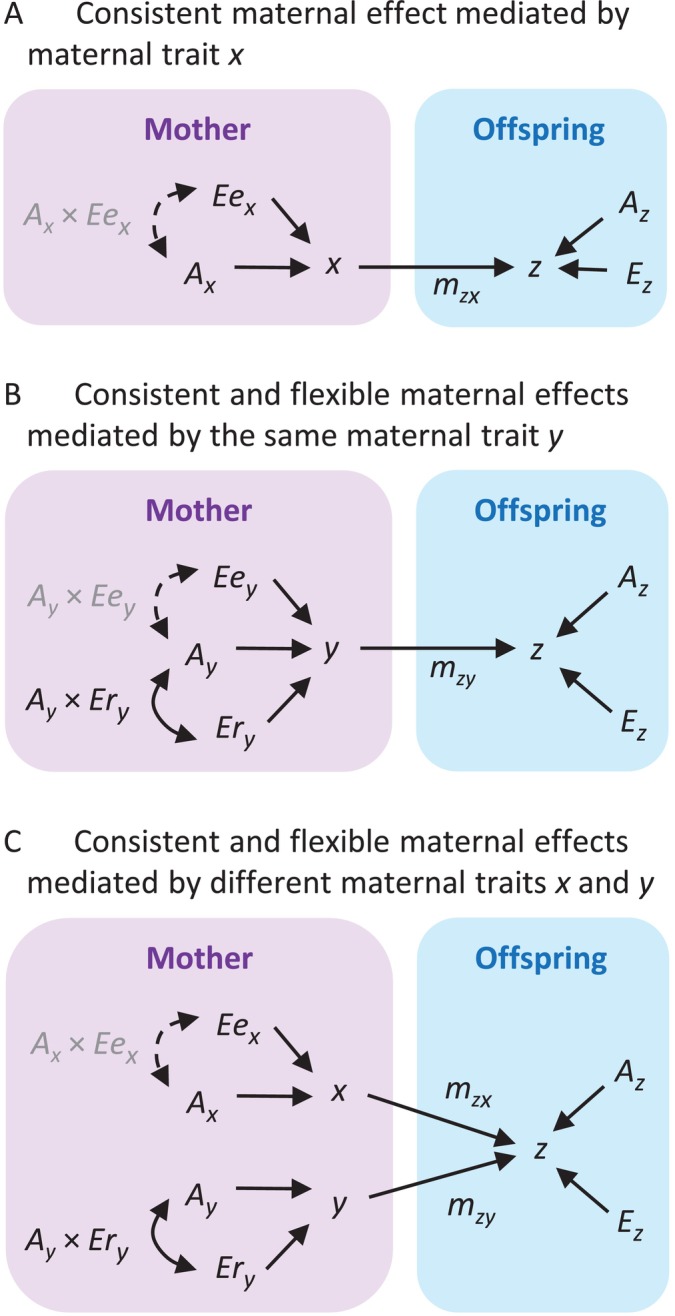
Combined path models for quantifying maternal trait variances in mothers and for testing maternal effects in offspring under different scenarios (adapted from McAdam *et al*., 2014). In model (A), a single maternal trait, *x*, mediates a consistent maternal effect on an offspring trait, *z*, and in model (B), another maternal trait, *y*, mediates both consistent and flexible maternal effects on *z*. Variations in *x* and *y* are partitioned into an additive genetic source (*A*), and early (*Ee*) and/or late (*Er*) environmental sources that affect the development of the maternal phenotype, respectively, during early life and during reproduction. In model (C), maternal effects on *z* are mediated by two different maternal traits, *x* and *y*, with different genetic and environmental sources of phenotypic variation. Phenotypic variations in *x* and *y* are determined by environmental differences experienced by females during early life and during reproductive life, respectively. The effects of different maternal traits on *z* are represented by *m*_*zx*_ and *m*_*zy*_. Double‐headed arrows indicate interactions between genetic and environmental effects, and dotted arrows are inconspicuous effects, not considered in the models presented in Section III.

Here, we conceptualise how genetic and environmental effects determine consistent and flexible maternal effect processes under different scenarios (Fig. 2; adapted from McAdam *et al*., 2014) and provide a quantitative genetic approach for empiricists. Consistent and flexible maternal effects can be quantified either simultaneously or separately, but here we consider the two maternal effects within the same framework. Depending on the goals of each study, the characteristics of available data, or experimental design, the framework provided here may be either reduced or extended. For example, if pedigree information is unavailable, an individual‐based approach without an additive genetic component can be used.

In our framework, we assume that maternal effects arise due to variations among females/genotypes (consistent maternal phenotype) and within females/genotypes (flexible maternal phenotype) in maternal traits that influence the offspring phenotype. These among‐ and within‐female variations arise from environmental effects across ontogeny, genetic effects, or G × E interaction. The latter shows differential performance of genotypes as a function of the environment where they are expressed, and it is especially important for the flexible maternal phenotype. In egg‐laying animals, for example, mothers affect offspring size at hatching through egg size. Under different scenarios, this maternal trait may be consistently variable among different females but largely persistent within the same females/genotypes (Fig. 2A) or may change across different environments within the same females/genotypes (Fig. 2B). The variation among females could be due to individual differences in the underlying genetics of egg size (*A*) and the environmental conditions experienced during early life (*Ee*) or during reproductive life (*Er*). Differences among females in how they respond to early‐life conditions could also be genetically determined, producing a G × E interaction (*A* × *Ee*) in consistent egg size variation. Although this *A* × *Ee* interaction is not considered in our model for simplification, *A* × *Ee* could be included in an extended model if the experimental design allows its quantification or if there are exhaustive data from multiple generations exposed to different early environmental conditions. How egg size changes within females in response to changing environmental conditions during their reproductive life can be tested by modelling the *A* × *Er* interaction.

We also consider another situation, where consistent and flexible maternal effects are mediated by two different maternal traits (Fig. 2C). For example, let us assume that offspring size at hatching can be affected by both egg size and maternal stress hormone transferred to the egg, which are maternal phenotypes influenced by environments experienced by the mother during her early life and reproductive life, respectively. Thus, among‐female variation in egg size and within‐female variation in maternal hormone level would affect offspring development through consistent and flexible maternal effect processes, respectively.

At the analytic level, we propose to examine maternal effects in two steps: first, by using a variance‐partitioning strategy to analyse phenotypic variations in maternal traits (i.e. ontogeny of the maternal phenotype), and then by using trait‐based strategies to test how these maternal traits affect the offspring (i.e. maternal effect outcome). Thus, both maternal and offspring traits should be measured to apply this two‐level method. These traits can be measured either once or multiple times across environments and time, and model composition will change depending on this as explained below. To develop our framework for studying consistent and flexible maternal effect processes, we apply the hybrid approach of variance partitioning and trait‐based test, previously described in McAdam *et al*. (2014) for measuring maternal effects. For this, we use both simple animal model and random regression (RR) animal model approaches. The Animal model is a form of mixed model in which phenotypic variation among individuals for a trait is partitioned into different components. In an animal model, the additive genetic variance is estimated based on a comparison of phenotypes of relatives (Wilson *et al*., 2010). A RR animal model is a form of animal model in which individual phenotypes are modelled as a continuous function of a covariate, which may be an environmental variable in studies of plasticity and G × E (Nussey, Wilson & Brommer, 2007). In a RR animal model, the intercepts and slopes of individuals' functions are fitted as random effects.

Here, we consider the scenario shown in Fig. 2C, where consistent and flexible maternal effects on an offspring trait, *z*, are mediated by two different maternal traits, *x* and *y*, but our models can be either reduced or extended, depending on the given scenario and available data set. We first model maternal trait *x*, involved in a consistent maternal effect process and expressed by individual mother *i*, in a simple animal model as:

(1) $$x_{i}=\mu^{x}+{a^{x}}_{i}+{\mathrm{ee}}_{i}+{{ɛ}^{x}}_{i}$$

where *μ* is the population mean of the trait, *a*_*i*_ is the additive genetic merit of individual *i*, *ee*_*i*_ is the early environmental effect, and *ɛ*_*i*_ is a homogeneous random residual error. In this mixed‐effect model, *μ* is included as a fixed effect, whereas all other components are random effects. In this simplest model, *ee*_*i*_ is a kind of common environmental effect (*ce*), such as cohort and experimental treatment, experienced by individual mothers during their early development, and it is assumed that the trait was measured only once per individual. In another situation, where the consistent maternal trait was repeatedly measured within individual females, the *ce* component can be replaced by the permanent environmental effect (*pe*) component in the model. Thus, model 1 can be modified as:

(2) $$x_{i}=\mu^{x}+{a^{x}}_{i}+{g^{x}}_{i}\left({\mathrm{ee}}_{i}\right)+{{ɛ}^{x}}_{i}$$

where *g*_*i*_(*ee*_*i*_) is the *pe* function (i.e. among‐individual difference) expressed in relation to the early environment experienced by individuals.

Then, we model maternal trait *y*, involved in a flexible maternal effect process and expressed by individual mother *i* at time or age *t*, in a RR animal model. For a simple illustration, here we develop the RR models in the context of a linear (i.e. first‐order) reaction norm, which is characterised by an elevation and slope (see also Nussey *et al*., 2007). Thus, the flexible maternal trait *y* is modelled as:

(3) $$y_{\mathrm{it}}=\mu^{y}+f\left({a^{y}}_{i},{\mathrm{er}}_{\mathrm{it}}\right)+f\left({g^{y}}_{i},{\mathrm{er}}_{\mathrm{it}}\right)+{{ɛ}^{y}}_{\mathrm{it}}$$

where *f*(*a*_*i*_, *er*_*it*_) is the RR function of additive genetic effect, which varies at the genetic level according to the reproductive environment *er* (i.e. genotype‐by‐reproductive environment interaction, *A* × *Er*), *f*(*g*_*i*_, *er*_*it*_) is the RR *pe* function (i.e. among‐individual difference in the interaction with reproductive environment), and *ɛ*_*it*_ is a homogeneous residual error. Thus, in model 3, we assume that the maternal trait changes within genotypes and females according to the reproductive environment, such as season, experimental treatment, and habitat. If the trait was measured only once per individual in a population study of genetically related individuals, the *pe* function can be excluded in the model, although in such a case the genetic effect estimate can be inflated. If multiple measurements per individual are available but there is no pedigree information, the *a* function can be excluded.

In the following step, we perform a trait‐based model simultaneously to test consistent and flexible maternal effects of *x* and *y* on offspring trait *z*, expressed by individual offspring *j* in a univariate animal model as:

(4) $$z_{j}=\mu^{z}+M_{x}+M_{y}+{a^{z}}_{j}+{m^{z}}_{j}+{{ɛ}^{z}}_{j}$$

where *M*_*x*_ and *M*_*y*_ are the two maternal traits explored in models 1–3 and included as fixed effects in this model to test directly their effects on the offspring trait. This model assumes that the offspring phenotype is measured only once per individual offspring, although multiple descendants from the same mother are included in the data set, as is the case for many empirical studies of maternal effects. An additional variance component *m*_*j*_ is included in this offspring‐trait model to estimate how much of their phenotypic variation is due to a maternal effect. This maternal effect variance can be estimated, for example, by including maternal identity as a random effect.

Model 4 can be further extended by including maternal variances related to the maternal traits *x* and *y* in a RR animal model, which describes the phenotype *z* of individual offspring *j* produced by mother *i* at time *t*, as:

(5) $$z_{\mathrm{ijt}}=\mu^{z}+M_{x}+M_{y}+{a^{z}}_{j}+{m^{\mathrm{zx}}}_{j}\left({\mathrm{ee}}_{i}\right)+f\left({m^{\mathrm{zy}}}_{j},{\mathrm{er}}_{\mathrm{it}}\right)+{{ɛ}^{z}}_{\mathrm{jt}}$$

where *m*_*j*_(*ee*_*i*_) is the consistent maternal environment effect function, explaining variance in *z* due to the early maternal environment, and *f*(*m*_*j*_, *er*_*it*_) is the RR function, describing the flexible maternal environment variance as a function of the reproductive environment. Thus, these are the maternal components of individual *j*'s reaction norm elevation in response to the early maternal environment, and reaction norm elevation and slope in response to the maternal reproductive environment.

In our models described here, we assumed only homogeneous residual errors and linear reaction norm slopes. However, the RR models can be modified to allow a heterogeneous residual variance as a function of the environment or to describe non‐linear reaction norm slopes (by using an orthogonal polynomial function). Analysing simultaneously maternal and offspring trait reaction norms across ontogeny is a challenging but necessary task to improve our understanding of different maternal effect processes and their mechanisms. We hope that our framework provides a guideline to design future empirical studies for the purpose of testing consistent and flexible maternal effect processes.

## IV. FUTURE PERSPECTIVES ON MATERNAL EFFECTS AND LIFE‐HISTORY DIVERSITY

By incorporating the ontogeny of the maternal phenotype and advanced quantitative genetic approaches into the study of maternal effect processes, our framework offers a robust toolset for exploring how different maternal reproductive strategies contribute to variation in life‐history trade‐offs across diverse ecological and evolutionary contexts.

The relative importance of consistent and flexible maternal effects in each species or population may depend on when and how mothers obtain resources for provisioning their offspring. Some species provision eggs and/or young using energy gained concurrently (i.e. income breeders), while others use energy stores accumulated at an earlier time [i.e. capital breeders (Bonnet, Bradshaw & Shine, 1998; Jönsson, 1997)]. Many studies have analysed the costs and benefits of accumulating capital or the relative trade‐offs in terms of current and future offspring (reviewed in Alonso‐Alvarez & Velando, 2012), but few have explicitly explored whether the ontogeny of the maternal phenotype and its interaction with the environment moulds the success of capital *versus* income reproductive strategies. Intuitive thinking tells us that flexible maternal effects could facilitate “income breeder” strategies, which depend on ongoing resource intake during the reproductive period to adapt offspring provisioning to fluctuating conditions (Amarillo‐Suárez & Fox, 2006). By contrast, when the environment provides a stable and efficient resource storage prior to reproduction, and probably already during development, the consistent component of maternal effects should be more relevant to guarantee the success of “capital breeder” strategies in females. One way to test these predictions would be to expose females experimentally to either changing or unchanging resources during early development and then track their maternal resource allocation and fitness throughout life. We expect that stable and high resource availability during early life will lead, on average, to higher consistent maternal resource allocation in the offspring (like in silver spoon effects), thus facilitating a lifelong capital strategy that is less dependent on the quality of the reproductive environment.

Such a buffering mechanism would mirror Waddington's idea of canalisation, where developmental systems are organised to absorb perturbations and maintain robustness in the face of external or internal fluctuations (Waddington, 1942). However, while there is strong evidence for canalisation as an evolved property that varies among genotypes, the developmental and genetic mechanisms that produce this phenomenon are very poorly understood (Hallgrimsson *et al*., 2019). Mothers may influence the phenotypic robustness of their offspring by supplying stable and consistent resources during critical stages of development, thereby ensuring a specific developmental trajectory despite environmental perturbations. For instance, maternal effects have been shown to contribute to the canalisation of germination timing in plants, where consistent maternal provisioning induces stronger dormancy in seeds, leading to more uniform germination patterns (Edwards *et al*., 2017). Hence, investigating how consistent maternal effects contribute to phenotypic stability can shed light on the mechanisms that promote developmental canalisation.

Importantly, maternal effect processes can change with maternal age. This is an interesting topic and there is scope for both consistent and flexible maternal effects, because both early developmental and later reproductive environments are likely to affect individual ageing patterns. In many animal taxa, individuals developing under early environments that are more benign usually experience later onsets and slower rates of reproductive senescence (Bouwhuis *et al*., 2010; Kim, Metcalfe & Velando, 2016; Sanghvi *et al*., 2021). Indeed, most published studies have focussed only on the influence of the early developmental environment on reproductive senescence, probably because early life stages are the most critical periods when available resources must be distributed between growth, first reproduction and somatic maintenance (Lemaître *et al*., 2015; Lemaître & Gaillard, 2017). Yet, the deficiency of concurrent resources can be expected to reinforce the detrimental effects of old maternal age (Oro *et al*., 2014; van den Heuvel, English & Uller, 2016). Therefore, studies on reproductive senescence that consider both the quality of developmental and reproductive environments are needed and would provide deeper insights into how maternal strategies contribute to the variation in life‐history trade‐offs across environments.

Moreover, in iteroparous animals, it is now well known that an advanced maternal age negatively affects not only the performance of the mothers themselves but also the lifespan and fertility of their offspring, a phenomenon that is named the “Lansing effect” (Lansing, 1947; Monaghan, Maklakov & Metcalfe, 2020). This occurs in many animal species, including humans, mainly due to maternal effect senescence (i.e. maternal age‐related decline in maternal traits), which evolves independently from reproductive senescence and results in age‐related declines in offspring birth mass, early growth or survival (Moorad & Nussey, 2016; Hernández *et al*., 2020). Yet, empirical evidence for maternal effect senescence remains scarce, although, in theory, it should be stronger than reproductive senescence (Lemaître & Gaillard, 2017; Moorad & Nussey, 2016). Furthermore, from oocyte production to maternal care, there are diverse potential mechanisms of maternal effects by which maternal age can affect offspring quality and viability. For example, maternal effect senescence may be mediated by reduced egg quality (Beamonte‐Barrientos *et al*., 2010), and by the capacity of eggs to detect and repair DNA damage, especially prior to embryonic genome activation (Kim *et al*., 2023). However, here the focus has mainly been on reproductive senescence, while the early developmental environment is likely to influence the Lansing effect and maternal effect senescence too. Hence, a remaining challenge for researchers is to consider how the different environments experienced by a female throughout ontogeny modulate the patterns of ageing and senescence (Shefferson, Jones & Salguero‐Gómez, 2017). This could be explored by means of crossover manipulations of the mother's developmental and reproductive environments, by exposing females to contrasting environments during early‐life development, followed by reproductive environments in either matched or mismatched conditions (Burton & Metcalfe, 2014; see also Section III). Then, by incorporating individual‐based random regression models, the individual × age interaction variation in responsiveness to environmental changes could be explicitly tested (Charmantier, Brommer & Nussey, 2014).

In our framework, the interaction between consistent and flexible maternal effects may provide the key to understanding the effects of a match/mismatch between the early and late maternal environments, and consequently, between the maternal and offspring environments. Therefore, the fitness effects of the early and late maternal environments should be explored jointly, since they can interactively influence a female's phenotype, which may have in turn additive, synergistic or antagonistic effects on the offspring phenotype depending on the environmental match (Bateson *et al*., 2004; Noguera & Velando, 2020). To test such interaction between different maternal effects, researchers could implement crossover manipulations of the mother's developmental and reproductive environments, as mentioned above, which should be followed by split‐brood experiments, in which the offspring born to the same mother are reared under contrasting conditions. Such experiments manipulating environmental conditions across maternal ontogenetic stages and generations and assessing fitness consequences can help to unveil whether consistent and flexible maternal effects interactively produce an adaptive offspring phenotype (e.g. fitness is highest when the early and late maternal environments are matched; Shea, Pen & Uller, 2011). Additionally, longitudinal studies on wild populations would offer complementary insights and are needed to examine the influence of naturally fluctuating conditions throughout ontogeny on maternal effect processes.

Another remaining question is which specific maternal traits are more likely to exert consistent or flexible maternal effects on the offspring. Early life conditions such as resource availability or social density may influence a mother's developmental plans and have a permanent influence on traits like her body size (Fox & Savalli, 1998), which in turn can determine egg size and nourishment. On the other hand, aspects of the reproductive environment (e.g. temperature and predation risk) have been found to influence the mother's reproductive timing and her physiology (Table 3). However, the mother's early environment can influence maternal physiology too and thus hormone or carotenoid allocation to eggs (Crocker & Hunter, 2018; Kim *et al*., 2022; Table 2). Thus, further studies could rather explore the relative importance of consistent or flexible maternal effects by acknowledging the ontogeny of maternal effect processes in experimental designs and implementing the combined approach of variance partitioning and trait‐based strategies (i.e. analysing the development of different maternal traits and their effects on the offspring; see Section III).

Maternal effects provide important mechanisms that facilitate the expression of novel and functional phenotypic traits in response to environmental changes and produce evolutionarily significant variation by genetic accommodation. Thus, acknowledging the differences between consistent and flexible maternal effect processes will improve our understanding of how phenotypic responses to environmental changes contribute to genetic changes for the next generations.

## V. CONCLUSIONS

(1) Maternal effects are key mechanisms underlying the gene–environment nexus, and the ways in which the environment experienced by a mother influence offspring development are extremely diverse.
(2) The major focus and interest of theoretical and empirical studies of maternal effects has been testing whether they are adaptive or not for the mother and the offspring. This mainstream outcome‐based approach has improved our understanding of the evolution of maternal effects, but we still know little about the causes and consequences of maternal effects.
(3) Empirical studies often produce contrasting results regarding different maternal effect hypotheses. This is, in part, because maternal traits are often not identified in the studies, and because their ontogeny has received relatively little attention compared to the offspring phenotype.
(4) Here, we propose a conceptual and methodological framework that distinguishes between consistent and flexible maternal effect processes, according to the ontogenetic stage at which a specific maternal trait is developed and how long its effect persists. Consistent maternal effects are mediated by the maternal phenotype developed mainly during the mother's early life, whereas flexible maternal effects are mediated by the maternal phenotype that changes in response to the environment experienced during the mother's reproductive life.
(5) Consistent and flexible maternal effects are likely involved in different maternal effect processes, the former mainly in condition‐transfer effects, silver spoon effects, cohort effects and cascading effects, while the latter in anticipatory effects, intergenerational plasticity, developmental programming and the Lansing effect, although some processes can be viewed under both perspectives. The ontogenetic scope of the maternal phenotype should help researchers to refine predictions of these maternal effect hypotheses, thereby increasing the possibility of successful hypothesis testing.
(6) We developed a quantitative genetic model approach for empiricists, which combines variance partitioning and trait‐based strategies, to test consistent and flexible maternal effect processes. This approach first determines how early and/or late environments shape the maternal phenotype across ontogeny, then examines how this maternal phenotype affects the offspring phenotype.
(7) The conceptual and methodological framework of consistent and flexible maternal effects should improve our understanding of how the environment–phenotype nexus mediates maternal effect processes and allow us rigorously to test outstanding questions of maternal effects and life‐history diversity in relation to, for example, resource acquisition and allocation, senescence, and the effects of environmental match/mismatch.

## References

[brv70062-bib-0001] Agrawal, A. A. , Laforsch, C. & Tollrian, R. (1999). Transgenerational induction of defences in animals and plants. Nature 401, 60–63.

[brv70062-bib-0002] Alonso‐Alvarez, C. & Velando, A. (2012). Benefits and costs of parental care. In The Evolution of Parental Care (eds N. J. Royle , P. T. Smiseth and M. Kölliker ), pp. 40–61. Oxford University Press, Oxford.

[brv70062-bib-0003] Amarillo‐Suárez, A. R. & Fox, C. W. (2006). Population differences in host use by a seed‐beetle: local adaptation, phenotypic plasticity and maternal effects. Oecologia 150, 247–258. 16915403 10.1007/s00442-006-0516-y

[brv70062-bib-0004] Badás, E. P. , Romero‐Haro, A. & Morales, J. (2025). What doesn't kill you makes you (and your descendants) stronger: early‐life exposure to human‐induced challenges as a trigger of compensatory mechanisms. Journal of Avian Biology 2025, e03418.

[brv70062-bib-0005] Badyaev, A. V. (2008). Maternal effects as generators of evolutionary change. Annals of the New York Academy of Sciences 1133, 151–161. 18559819 10.1196/annals.1438.009

[brv70062-bib-0006] Badyaev, A. V. & Uller, T. (2009). Parental effects in ecology and evolution: mechanisms, processes and implications. Philosophical Transactions of the Royal Society B: Biological Sciences 364, 1169–1177. 10.1098/rstb.2008.0302 PMC2666689 19324619

[brv70062-bib-0007] Bateson, P. , Barker, D. , Clutton‐Brock, T. , Deb, D. , D'Udine, B. , Foley, R. A. , Gluckman, P. , Godfrey, K. , Kirkwood, T. , Mirazón Lahr, M. , McNamara, J. , Metcalfe, N. B. , Monaghan, P. , Spencer, H. G. & Sultan, S. E. (2004). Developmental plasticity and human health. Nature 430, 419–421. 15269759 10.1038/nature02725

[brv70062-bib-0008] Bauerfeind, S. S. & Fischer, K. (2005). Effects of food stress and density in different life stages on reproduction in a butterfly. Oikos 111, 514–524.

[brv70062-bib-0009] Beamonte‐Barrientos, R. , Velando, A. , Drummond, H. & Torres, R. (2010). Senescence of maternal effects: aging influences egg quality and rearing capacities of a long‐lived bird. American Naturalist 175, 469–480. 10.1086/650726 20175680

[brv70062-bib-0010] Beckerman, A. , Benton, T. G. , Ranta, E. , Kaitala, V. & Lundberg, P. (2002). Population dynamic consequences of delayed life‐history effects. Trends in Ecology & Evolution 17, 263–269.

[brv70062-bib-0011] Bodden, C. , Hannan, A. J. & Reichelt, A. C. (2020). Diet‐induced modification of the sperm epigenome programs metabolism and behavior. Trends in Endocrinology & Metabolism 31, 131–149. 31744784 10.1016/j.tem.2019.10.005

[brv70062-bib-0012] Bonduriansky, R. (2021). Plasticity across generations. In Phenotypic Plasticity & Evolution (ed. D. W. Pfennig ), pp. 327–348. CRC Press, Boca Raton.

[brv70062-bib-0013] Bonduriansky, R. & Crean, A. J. (2018). What are parental condition‐transfer effects and how can they be detected? Methods in Ecology and Evolution 9, 450–456.

[brv70062-bib-0014] Bonnet, T. , Morrissey, M. B. , de Villemereuil, P. , Alberts, S. C. , Arcese, P. , Bailey, L. D. , Boutin, S. , Brekke, P. , Brent, L. J. & Camenisch, G. (2022). Genetic variance in fitness indicates rapid contemporary adaptive evolution in wild animals. Science 376, 1012–1016. 35617403 10.1126/science.abk0853

[brv70062-bib-0015] Bonnet, X. , Bradshaw, D. & Shine, R. (1998). Capital versus income breeding: an ectothermic perspective. Oikos 83, 333–342.

[brv70062-bib-0016] Boulinier, T. & Staszewski, V. (2008). Maternal transfer of antibodies: raising immuno‐ecology issues. Trends in Ecology & Evolution 23, 282–288. 18375011 10.1016/j.tree.2007.12.006

[brv70062-bib-0017] Bouwhuis, S. , Charmantier, A. , Verhulst, S. & Sheldon, B. C. (2010). Individual variation in rates of senescence: natal origin effects and disposable soma in a wild bird population. Journal of Animal Ecology 79, 1251–1261. 20646122 10.1111/j.1365-2656.2010.01730.x

[brv70062-bib-0018] Brommer, J. E. , Merilä, J. , Sheldon, B. C. & Gustafsson, L. (2005). Natural selection and genetic variation for reproductive reaction norms in a wild bird population. Evolution 59, 1362–1371. 16050111

[brv70062-bib-0019] Brommer, J. E. , Pietiäinen, H. & Kolunen, H. (2003). Natural selection on individual clutch size‐laying date trends in the Ural owl. Evolutionary Ecology Research 5, 229–237.

[brv70062-bib-0020] Burgess, S. C. & Marshall, D. J. (2014). Adaptive parental effects: the importance of estimating environmental predictability and offspring fitness appropriately. Oikos 123, 769–776.

[brv70062-bib-0021] Burton, T. & Metcalfe, N. B. (2014). Can environmental conditions experienced in early life influence future generations? Proceedings of the Royal Society B: Biological Sciences 281, 20140311. 10.1098/rspb.2014.0311 PMC4024293 24807254

[brv70062-bib-0022] Castano‐Sanz, V. , Gomez‐Mestre, I. & Garcia‐Gonzalez, F. (2022). Evolutionary consequences of pesticide exposure include transgenerational plasticity and potential terminal investment transgenerational effects. Evolution 76, 2649–2668. 36117275 10.1111/evo.14613

[brv70062-bib-0023] Champagne, F. A. , Francis, D. D. , Mar, A. & Meaney, M. J. (2003). Variations in maternal care in the rat as a mediating influence for the effects of environment on development. Physiology & Behavior 79, 359–371. 12954431 10.1016/s0031-9384(03)00149-5

[brv70062-bib-0024] Champagne, F. A. & Meaney, M. J. (2007). Transgenerational effects of social environment on variations in maternal care and behavioral response to novelty. Behavioral Neuroscience 121, 1353–1363. 18085888 10.1037/0735-7044.121.6.1353

[brv70062-bib-0025] Charmantier, A. , Brommer, J. E. & Nussey, D. H. (2014). The quantitative genetics of senescence in wild animals. In Quantitative Genetics in the Wild (eds A. Charmantier , D. Garant and L. E. B. Kruuk ), pp. 68–83. Oxford University Press, Oxford.

[brv70062-bib-0026] Charmantier, A. , McCleery, R. H. , Cole, L. R. , Perrins, C. , Kruuk, L. E. & Sheldon, B. C. (2008). Adaptive phenotypic plasticity in response to climate change in a wild bird population. Science 320, 800–803. 18467590 10.1126/science.1157174

[brv70062-bib-0027] Crean, A. J. & Bonduriansky, R. (2014). What is a paternal effect? Trends in Ecology & Evolution 29, 554–559. 25130305 10.1016/j.tree.2014.07.009

[brv70062-bib-0028] Crean, A. J. & Marshall, D. J. (2009). Coping with environmental uncertainty: dynamic bet hedging as a maternal effect. Philosophical Transactions of the Royal Society B 364, 1087–1096. 10.1098/rstb.2008.0237 PMC2666679 19324613

[brv70062-bib-0029] Crocker, K. C. & Hunter, M. D. (2018). Environmental causes and transgenerational consequences of ecdysteroid hormone provisioning in *Acheta domesticus* . Journal of Insect Physiology 109, 69–78. 29890170 10.1016/j.jinsphys.2018.06.003

[brv70062-bib-0030] Donelson, J. M. , Munday, P. L. , McCormick, M. I. & Pitcher, C. R. (2012). Rapid transgenerational acclimation of a tropical reef fish to climate change. Nature Climate Change 2, 30–32.

[brv70062-bib-0031] Douhard, M. , Gaillard, J.‐M. , Delorme, D. , Capron, G. , Duncan, P. , Klein, F. & Bonenfant, C. (2013). Variation in adult body mass of roe deer: early environmental conditions influence early and late body growth of females. Ecology 94, 1805–1814. 24015524 10.1890/13-0034.1

[brv70062-bib-0032] Edwards, B. , Burghardt, L. T. , Kovach, K. E. & Donohue, K. (2017). Canalization of seasonal phenology in the presence of developmental variation: seed dormancy cycling in an annual weed. Integrative and Comparative Biology 57, 1021–1039. 28992196 10.1093/icb/icx065

[brv70062-bib-0033] Engqvist, L. & Reinhold, K. (2016). Adaptive trans‐generational phenotypic plasticity and the lack of an experimental control in reciprocal match/mismatch experiments. Methods in Ecology and Evolution 7, 1482–1488.

[brv70062-bib-0034] Fox, C. W. & Savalli, U. M. (1998). Inheritance of environmental variation in body size: superparasitism of seeds affects progeny and grandprogeny body size via a nongenetic maternal effect. Evolution 52, 172–182. 28568152 10.1111/j.1558-5646.1998.tb05150.x

[brv70062-bib-0035] Fox, C. W. , Thakar, M. S. & Mousseau, T. A. (1997). Egg size plasticity in a seed beetle: an adaptive maternal effect. American Naturalist 149, 149–163.

[brv70062-bib-0036] Francis, D. , Diorio, J. , Liu, D. & Meaney, M. J. (1999). Nongenomic transmission across generations of maternal behavior and stress responses in the rat. Science 286, 1155–1158. 10550053 10.1126/science.286.5442.1155

[brv70062-bib-0037] Galloway, L. F. & Etterson, J. R. (2007). Transgenerational plasticity is adaptive in the wild. Science 318, 1134–1136. 18006745 10.1126/science.1148766

[brv70062-bib-0038] Gauzere, J. , Pemberton, J. M. , Morris, S. , Morris, A. , Kruuk, L. E. & Walling, C. A. (2020). The genetic architecture of maternal effects across ontogeny in the red deer. Evolution 74, 1378–1391. 32462712 10.1111/evo.14000

[brv70062-bib-0039] Gauzere, J. , Walling, C. A. , Pick, J. L. , Watt, K. , Jack, P. , Morris, A. , Morris, S. & Pemberton, J. M. (2021). The role of maternally transferred antibodies in maternal performance in red deer. Ecology Letters 24, 2065–2076. 34245475 10.1111/ele.13834

[brv70062-bib-0040] Giesing, E. R. , Suski, C. D. , Warner, R. E. & Bell, A. M. (2011). Female sticklebacks transfer information via eggs: effects of maternal experience with predators on offspring. Proceedings of the Royal Society B: Biological Sciences 278, 1753–1759. 10.1098/rspb.2010.1819 PMC3081764 21068041

[brv70062-bib-0041] Grafen, A. (1988). In On the Uses of Data on Lifetime Reproductive Success. In Reproductive Success: Studies of Individual Variation in Contrasting Breeding Systems (ed. T. H. Clutton‐Brock ), pp. 454–471. University Chicago Press, Chicago.

[brv70062-bib-0042] Groothuis, T. G. G. , Hsu, B.‐Y. , Kumar, N. & Tschirren, B. (2019). Revisiting mechanisms and functions of prenatal hormone‐mediated maternal effects using avian species as a model. Philosophical Transactions of the Royal Society B: Biological Sciences 374, 20180115. 10.1098/rstb.2018.0115 PMC6460091 30966885

[brv70062-bib-0043] Hadfield, J. (2012). The quantitative genetic theory of parental effects. In The Evolution of Parental Care (eds N. J. Royle , P. T. Smiseth and M. Kölliker ), pp. 267–284. Oxford University Press, Oxford.

[brv70062-bib-0044] Hallgrimsson, B. , Green, R. M. , Katz, D. C. , Fish, J. L. , Bernier, F. P. , Roseman, C. C. , Young, N. M. , Cheverud, J. M. & Marcucio, R. S. (2019). The developmental‐genetics of canalization. Seminars in Cell & Developmental Biology 88, 67–79. 29782925 10.1016/j.semcdb.2018.05.019 PMC6251770

[brv70062-bib-0045] Helle, H. , Koskela, E. & Mappes, T. (2012). Life in varying environments: experimental evidence for delayed effects of juvenile environment on adult life history. Journal of Animal Ecology 81, 573–582. 22191455 10.1111/j.1365-2656.2011.01937.x

[brv70062-bib-0046] Hernández, C. M. , van Daalen, S. F. , Caswell, H. , Neubert, M. G. & Gribble, K. E. (2020). A demographic and evolutionary analysis of maternal effect senescence. Proceedings of the National Academy of Sciences 117, 16431–16437. 10.1073/pnas.1919988117 PMC7368264 32601237

[brv70062-bib-0047] Hopwood, P. E. , Moore, A. J. & Royle, N. J. (2014). Effects of resource variation during early life and adult social environment on contest outcomes in burying beetles: a context‐dependent silver spoon strategy? Proceedings of the Royal Society B: Biological Sciences 281, 20133102. 10.1098/rspb.2013.3102 PMC4024278 24789890

[brv70062-bib-0048] Horton, T. H. & Stetson, M. H. (1990). Maternal programming of the fetal brain dictates the response of juvenile Siberian hamsters to photoperiod: dissecting the information transfer system. Journal of Experimental Zoology 256, 200–202. 1974794 10.1002/jez.1402560443

[brv70062-bib-0049] Houston, A. I. , Stephens, P. A. , Boyd, I. L. , Harding, K. C. & McNamara, J. M. (2006). Capital or income breeding? A theoretical model of female reproductive strategies. Behavioral Ecology 18, 241–250.

[brv70062-bib-0050] Hsu, B.‐Y. , Verhagen, I. , Gienapp, P. , Darras, V. M. , Visser, M. E. & Ruuskanen, S. (2019). Between‐and within‐individual variation of maternal thyroid hormone deposition in wild great tits (*Parus major*). American Naturalist 194, E96–E108. 10.1086/704738 31490720

[brv70062-bib-0051] Jonsson, B. & Jonsson, N. (2014). Early environment influences later performance in fishes. Journal of Fish Biology 85, 151–188. 24961386 10.1111/jfb.12432

[brv70062-bib-0052] Jönsson, K. I. (1997). Capital and income breeding as alternative tactics of resource use in reproduction. Oikos 78, 57–66.

[brv70062-bib-0053] Kim, S.‐Y. , Álvarez‐Quintero, N. , Chiara, V. , Da Silva, A. & Velando, A. (2023). Maternal effect senescence via reduced DNA repair ability in the three‐spined stickleback. Molecular Ecology 32, 4648–4659. 37291748 10.1111/mec.17046

[brv70062-bib-0054] Kim, S.‐Y. , Chiara, V. , Álvarez‐Quintero, N. & Velando, A. (2022). Mitochondrial DNA content in eggs as a maternal effect. Proceedings of the Royal Society B: Biological Sciences 289, 20212100. 10.1098/rspb.2021.2100 PMC8767187 35042411

[brv70062-bib-0055] Kim, S.‐Y. , Metcalfe, N. B. , da Silva, A. & Velando, A. (2017). Thermal conditions during early life influence seasonal maternal strategies in the three‐spined stickleback. BMC Ecology 17, 34. 29126411 10.1186/s12898-017-0144-x PMC5681783

[brv70062-bib-0056] Kim, S.‐Y. , Metcalfe, N. B. & Velando, A. (2016). A benign juvenile environment reduces the strength of antagonistic pleiotropy and genetic variation in the rate of senescence. Journal of Animal Ecology 85, 705–714. 26559495 10.1111/1365-2656.12468 PMC4991295

[brv70062-bib-0057] Kruuk, L. E. (2004). Estimating genetic parameters in natural populations using the “animal model”. Philosophical Transactions of the Royal Society B: Biological Sciences 359, 873–890. 10.1098/rstb.2003.1437 PMC1693385 15306404

[brv70062-bib-0058] Lala, K. N. , Uller, T. , Feiner, N. , Feldman, M. W. & Gilbert, S. F. (2024). Evolution Evolving: The Developmental Origins of Adaptation and Biodiversity. Princeton, NJ: Princeton University Press.

[brv70062-bib-0059] Lansing, A. I. (1947). A transmissible, cumulative, and reversible factor in aging. Journal of Gerontology 2, 228–239. 20265000 10.1093/geronj/2.3.228

[brv70062-bib-0060] Lemaître, J.‐F. , Berger, V. , Bonenfant, C. , Douhard, M. , Gamelon, M. , Plard, F. & Gaillard, J.‐M. (2015). Early‐late life trade‐offs and the evolution of ageing in the wild. Proceedings of the Royal Society B: Biological Sciences 282, 20150209. 10.1098/rspb.2015.0209 PMC4426628 25833848

[brv70062-bib-0061] Lemaître, J. F. & Gaillard, J. M. (2017). Reproductive senescence: new perspectives in the wild. Biological Reviews 92, 2182–2199. 28374548 10.1111/brv.12328

[brv70062-bib-0062] Levis, N. A. & Pfennig, D. W. (2016). Evaluating ‘plasticity‐first’ evolution in nature: key criteria and empirical approaches. Trends in Ecology & Evolution 31, 563–574. 27067134 10.1016/j.tree.2016.03.012

[brv70062-bib-0063] Levis, N. A. & Pfennig, D. W. (2020). Plasticity‐led evolution: a survey of developmental mechanisms and empirical tests. Evolution & Development 22, 71–87. 31449722 10.1111/ede.12309

[brv70062-bib-0064] Lind, M. I. , Zwoinska, M. K. , Andersson, J. , Carlsson, H. , Krieg, T. , Larva, T. & Maklakov, A. A. (2020). Environmental variation mediates the evolution of anticipatory parental effects. Evolution Letters 4, 371–381. 32774885 10.1002/evl3.177 PMC7403678

[brv70062-bib-0065] Lindström, J. (1999). Early development and fitness in birds and mammals. Trends in Ecology & Evolution 14, 343–348. 10441307 10.1016/s0169-5347(99)01639-0

[brv70062-bib-0066] Lindström, J. & Kokko, H. (2002). Cohort effects and population dynamics. Ecology Letters 5, 338–344.

[brv70062-bib-0067] Lummaa, V. & Clutton‐Brock, T. (2002). Early development, survival and reproduction in humans. Trends in Ecology & Evolution 17, 141–147.

[brv70062-bib-0068] Marlow, F. L. (2020). Maternal Effect Genes in Development. Academic Press, Cambridge, MA.

[brv70062-bib-0069] Marquis, O. , Massot, M. & Le Galliard, J. F. (2008). Intergenerational effects of climate generate cohort variation in lizard reproductive performance. Ecology 89, 2575–2583. 18831178 10.1890/07-1211.1

[brv70062-bib-0070] Marshall, D. J. (2008). Transgenerational plasticity in the sea: context‐dependent maternal effects across the life history. Ecology 89, 418–427. 18409431 10.1890/07-0449.1

[brv70062-bib-0071] Marshall, D. J. & Uller, T. (2007). When is a maternal effect adaptive? Oikos 116, 1957–1963.

[brv70062-bib-0072] McAdam, A. G. , Garant, D. & Wilson, A. J. (2014). The effects of others' genes: maternal and other indirect genetic effects. In Quantitative Genetics in the Wild (eds A. Charmantier , D. Garant and L. E. Kruuk ), pp. 84–103. Oxford University Press, Oxford.

[brv70062-bib-0073] McGlothlin, J. W. & Galloway, L. F. (2014). The contribution of maternal effects to selection response: an empirical test of competing models. Evolution 68, 549–558. 24099096 10.1111/evo.12235

[brv70062-bib-0074] McJunkin, K. (2018). Maternal effects of microRNAs in early embryogenesis. RNA Biology 15, 165–169. 29120257 10.1080/15476286.2017.1402999 PMC5798946

[brv70062-bib-0075] Monaghan, P. (2008). Early growth conditions, phenotypic development and environmental change. Philosophical Transactions of the Royal Society B 363, 1635–1645. 10.1098/rstb.2007.0011 PMC2606729 18048301

[brv70062-bib-0076] Monaghan, P. , Maklakov, A. A. & Metcalfe, N. B. (2020). Intergenerational transfer of ageing: parental age and offspring lifespan. Trends in Ecology & Evolution 35, 927–937. 32741650 10.1016/j.tree.2020.07.005

[brv70062-bib-0077] Moorad, J. A. & Nussey, D. H. (2016). Evolution of maternal effect senescence. Proceedings of the National Academy of Sciences 113, 362–367. 10.1073/pnas.1520494113 PMC4720302 26715745

[brv70062-bib-0078] Moore, M. P. , Whiteman, H. H. & Martin, R. A. (2019). A mother's legacy: the strength of maternal effects in animal populations. Ecology Letters 22, 1620–1628. 31353805 10.1111/ele.13351

[brv70062-bib-0079] Morales, J. (2020). Eggshell biliverdin as an antioxidant maternal effect: biliverdin as an antioxidant resource in oviparous animals. BioEssays 42, 2000010. 10.1002/bies.202000010 32608113

[brv70062-bib-0080] Morrongiello, J. R. , Bond, N. R. , Crook, D. A. & Wong, B. B. M. (2012). Spatial variation in egg size and egg number reflects trade‐offs and bet‐hedging in a freshwater fish. Journal of Animal Ecology 81, 806–817. 22309288 10.1111/j.1365-2656.2012.01961.x

[brv70062-bib-0081] Mousseau, T. A. & Fox, C. W. (1998a). Maternal Effects as Adaptations. New York: Oxford University Press.

[brv70062-bib-0082] Mousseau, T. A. & Fox, C. W. (1998b). The adaptive significance of maternal effects. Trends in Ecology & Evolution 13, 403–407. 21238360 10.1016/s0169-5347(98)01472-4

[brv70062-bib-0083] Mousseau, T. A. , Uller, T. , Wapstra, E. & Badyaev, A. V. (2009). Evolution of maternal effects: past and present. Philosophical Transactions of the Royal Society B: Biological Sciences 364, 1035–1038. 10.1098/rstb.2008.0303 PMC2666690 19324608

[brv70062-bib-0084] Müller, G. B. (2007). Evo–devo: extending the evolutionary synthesis. Nature Reviews Genetics 8, 943–949. 10.1038/nrg2219 17984972

[brv70062-bib-0085] Noble, D. W. , McFarlane, S. E. , Keogh, J. S. & Whiting, M. J. (2014). Maternal and additive genetic effects contribute to variation in offspring traits in a lizard. Behavioral Ecology 25, 633–640.

[brv70062-bib-0086] Noguera, J. C. & Velando, A. (2020). Gull chicks grow faster but lose telomeres when prenatal cues mismatch the real presence of sibling competitors. Proceedings of the Royal Society B: Biological Sciences 287, 20200242. 10.1098/rspb.2020.0242 PMC7287347 32429809

[brv70062-bib-0087] Nussey, D. H. , Clutton‐Brock, T. H. , Elston, D. A. , Albon, S. D. & Kruuk, L. E. (2005a). Phenotypic plasticity in a maternal trait in red deer. Journal of Animal Ecology 74, 387–396.

[brv70062-bib-0088] Nussey, D. H. , Postma, E. , Gienapp, P. & Visser, M. E. (2005 *b*). Selection on heritable phenotypic plasticity in a wild bird population. Science 310, 304–306. 16224020 10.1126/science.1117004

[brv70062-bib-0089] Nussey, D. H. , Wilson, A. J. & Brommer, J. E. (2007). The evolutionary ecology of individual phenotypic plasticity in wild populations. Journal of Evolutionary Biology 20, 831–844. 17465894 10.1111/j.1420-9101.2007.01300.x

[brv70062-bib-0090] Oro, D. , Hernández, N. , Jover, L. & Genovart, M. (2014). From recruitment to senescence: food shapes the age‐dependent pattern of breeding performance in a long‐lived bird. Ecology 95, 446–457. 24669737 10.1890/13-0331.1

[brv70062-bib-0091] Osorno, J. L. , Morales, J. , Moreno, J. , Merino, S. , Tomás, G. & Vásquez, R. A. (2006). Evidence for differential maternal allocation to eggs in relation to manipulated male attractiveness in the pied flycatcher (*Ficedula hypoleuca*). Journal of Ornithology 147, 605–611.

[brv70062-bib-0092] Parsons, K. J. , Concannon, M. , Navon, D. , Wang, J. , Ea, I. , Groveas, K. , Campbell, C. & Albertson, R. C. (2016). Foraging environment determines the genetic architecture and evolutionary potential of trophic morphology in cichlid fishes. Molecular Ecology 25, 6012–6023. 27516345 10.1111/mec.13801

[brv70062-bib-0093] Parsons, K. J. , McWhinnie, K. , Pilakouta, N. & Walker, L. (2020). Does phenotypic plasticity initiate developmental bias? Evolution & Development 22, 56–70. 31348849 10.1111/ede.12304 PMC7004013

[brv70062-bib-0094] Pei, Y. , Forstmeier, W. & Kempenaers, B. (2020). Offspring performance is well buffered against stress experienced by ancestors. Evolution 74, 1525–1539. 32463119 10.1111/evo.14026

[brv70062-bib-0095] Petelle, M. B. , Dang, B. N. & Blumstein, D. T. (2017). The effect of maternal glucocorticoid levels on juvenile docility in yellow‐bellied marmots. Hormones and Behavior 89, 86–91. 28062231 10.1016/j.yhbeh.2016.12.014

[brv70062-bib-0096] Pfennig, D. W. (2021). Phenotypic Plasticity & Evolution. CRC Press, Boca Raton, FL.

[brv70062-bib-0097] Pfennig, D. W. & Martin, R. A. (2009). A maternal effect mediates rapid population divergence and character displacement in spadefoot toads. Evolution 63, 898–909. 19154374 10.1111/j.1558-5646.2008.00544.x

[brv70062-bib-0098] Pick, J. L. , Ebneter, C. , Hutter, P. & Tschirren, B. (2016). Disentangling genetic and prenatal maternal effects on offspring size and survival. American Naturalist 188, 628–639. 10.1086/688918 27860503

[brv70062-bib-0099] Pick, J. L. , Postma, E. & Tschirren, B. (2019). The more you get, the more you give: positive cascading effects shape the evolutionary potential of prenatal maternal investment. Evolution Letters 3, 412–423. 31388450 10.1002/evl3.125 PMC6675147

[brv70062-bib-0100] Piersma, T. & Drent, J. (2003). Phenotypic flexibility and the evolution of organismal design. Trends in Ecology & Evolution 18, 228–233.

[brv70062-bib-0101] Pigliucci, M. (2001). Phenotypic Plasticity: Beyond Nature and Nurture. John Hopkins University Press, Baltimore, MD.

[brv70062-bib-0102] Pruett, J. E. , Fargevieille, A. & Warner, D. A. (2020). Temporal variation in maternal nest choice and its consequences for lizard embryos. Behavioral Ecology 31, 902–910.

[brv70062-bib-0103] Qvarnström, A. & Price, T. D. (2001). Maternal effects, paternal effects and sexual selection. Trends in Ecology & Evolution 16, 95–100. 11165708 10.1016/s0169-5347(00)02063-2

[brv70062-bib-0104] Radersma, R. , Hegg, A. , Noble, D. W. A. & Uller, T. (2018). Timing of maternal exposure to toxic cyanobacteria and offspring fitness in *Daphnia magna*: implications for the evolution of anticipatory maternal effects. Ecology & Evolution 8, 12727–12736. 30619577 10.1002/ece3.4700 PMC6309005

[brv70062-bib-0105] Räsänen, K. & Kruuk, L. E. B. (2007). Maternal effects and evolution at ecological time‐scales. Functional Ecology 21, 408–421.

[brv70062-bib-0106] Räsänen, K. , Laurila, A. & Merilä, J. (2003a). Geographic variation in acid stress tolerance of the moor frog, *Rana arvalis*. I. Local adaptation. Evolution 57, 352–362. 12683531

[brv70062-bib-0107] Räsänen, K. , Laurila, A. & Merilä, J. (2003b). Geographic variation in acid stress tolerance of the moor frog, *Rana arvalis*. II. Adaptive maternal effects. Evolution 57, 363–371. 12683532

[brv70062-bib-0108] Reale, D. , McAdam, A. G. , Boutin, S. & Berteaux, D. (2003). Genetic and plastic responses of a northern mammal to climate change. Proceedings of the Royal Society B 270, 591–596. 12769458 10.1098/rspb.2002.2224 PMC1691280

[brv70062-bib-0109] Reed, T. E. , Wanless, S. , Harris, M. P. , Frederiksen, M. , Kruuk, L. E. & Cunningham, E. J. (2006). Responding to environmental change: plastic responses vary little in a synchronous breeder. Proceedings of the Royal Society B: Biological Sciences 273, 2713–2719. 10.1098/rspb.2006.3631 PMC1635500 17015329

[brv70062-bib-0110] Rodríguez‐Ruiz, G. , López, P. & Martín, J. (2020). Dietary vitamin D in female rock lizards induces condition‐transfer effects in their offspring. Behavioral Ecology 31, 633–640.

[brv70062-bib-0111] Roff, D. A. (1997). Evolutionary Quantitative Genetics. Chapman & Hall, New York, NY.

[brv70062-bib-0112] Roff, D. A. & Wilson, A. J. (2014). Quantifying genotype‐by‐environment interactions in laboratory systems. In Genotype‐by‐Environment Interactions and Sexual Selection (eds J. Hunt and D. Hosken ), pp. 101–136. John Wiley & Sons, Chichester.

[brv70062-bib-0113] Royle, N. J. , Surai, P. & Hartley, I. R. (2001). Maternally derived androgens and antioxidants in bird eggs: complementary but opposing effects? Behavioral Ecology 12, 381–385.

[brv70062-bib-0114] Salinas, S. & Munch, S. B. (2012). Thermal legacies: transgenerational effects of temperature on growth in a vertebrate. Ecology Letters 15, 159–163. 22188553 10.1111/j.1461-0248.2011.01721.x

[brv70062-bib-0115] Sanghvi, K. , Zajitschek, F. , Iglesias‐Carrasco, M. & Head, M. L. (2021). Sex‐ and trait‐specific silver‐spoon effects of developmental environments, on ageing. Evolutionary Ecology 35, 367–385.

[brv70062-bib-0116] Shama, L. N. S. , Strobel, A. , Mark, F. C. , Wegner, K. M. & Marshall, D. (2014). Transgenerational plasticity in marine sticklebacks: maternal effects mediate impacts of a warming ocean. Functional Ecology 28, 1482–1493.

[brv70062-bib-0117] Sharda, S. , Zuest, T. , Erb, M. & Taborsky, B. (2021). Predator‐induced maternal effects determine adaptive antipredator behaviors via egg composition. Proceedings of the National Academy of Sciences of the United States of America 118, e2017063118. 34507981 10.1073/pnas.2017063118 PMC8449421

[brv70062-bib-0118] Shea, N. , Pen, I. & Uller, T. (2011). Three epigenetic information channels and their different roles in evolution. Journal of Evolutionary Biology 24, 1178–1187. 21504495 10.1111/j.1420-9101.2011.02235.x PMC3116147

[brv70062-bib-0119] Shefferson, R. P. , Jones, O. R. & Salguero‐Gómez, R. (2017). The Evolution of Senescence in the Tree of Life. Cambridge University Press, Cambridge.

[brv70062-bib-0120] Smith, C. C. & Fretwell, S. D. (1974). The optimal balance between size and number of offspring. American Naturalist 108, 499–506.

[brv70062-bib-0121] Steiger, S. (2013). Bigger mothers are better mothers: disentangling size‐related prenatal and postnatal maternal effects. Proceedings of the Royal Society B: Biological Sciences 280, 20131225. 10.1098/rspb.2013.1225 PMC3730594 23843390

[brv70062-bib-0122] Strickland, K. , Mann, J. , Foroughirad, V. , Levengood, A. L. & Frère, C. H. (2021). Maternal effects and fitness consequences of individual variation in bottlenose dolphins' ecological niche. Journal of Animal Ecology 90, 1948–1960. 33942312 10.1111/1365-2656.13513

[brv70062-bib-0123] Sultan, S. (1996). Phenotypic plasticity for offspring traits in *Polygonum persicaria* . Ecology 77, 1791–1807.

[brv70062-bib-0124] Sultan, S. E. (2021). Phenotypic plasticity as an intrinsic property of organisms. In Phenotypic Plasticity & Evolution (ed. D. W. Pfennig ), pp. 3–24. CRC Press, Boca Raton, FL.

[brv70062-bib-0125] Taborsky, B. (2006 *a*). Mothers determine offspring size in response to own juvenile growth conditions. Biology Letters 2, 225–228. 17148368 10.1098/rsbl.2005.0422 PMC1618922

[brv70062-bib-0126] Taborsky, B. (2006 *b*). The influence of juvenile and adult environments on life‐history trajectories. Proceedings of the Royal Society B: Biological Sciences 273, 741–750. 10.1098/rspb.2005.3347 PMC1560075 16608695

[brv70062-bib-0127] Tomar, A. , Gomez‐Velazquez, M. , Gerlini, R. , Comas‐Armangué, G. , Makharadze, L. , Kolbe, T. , Boersma, A. , Dahlhoff, M. , Burgstaller, J. P. , Lassi, M. & Darr, J. (2024). Epigenetic inheritance of diet‐induced and sperm‐borne mitochondrial RNAs. Nature 630, 720–727. 38839949 10.1038/s41586-024-07472-3 PMC11186758

[brv70062-bib-0128] Tschirren, B. , Sendecka, J. , Groothuis, T. G. , Gustafsson, L. & Doligez, B. (2009). Heritable variation in maternal yolk hormone transfer in a wild bird population. American Naturalist 174, 557–564. 10.1086/605379 19737108

[brv70062-bib-0129] Uller, T. (2008). Developmental plasticity and the evolution of parental effects. Trends in Ecology & Evolution 23, 432–438. 18586350 10.1016/j.tree.2008.04.005

[brv70062-bib-0130] Uller, T. , Nakagawa, S. & English, S. (2013). Weak evidence for anticipatory parental effects in plants and animals. Journal of Evolutionary Biology 26, 2161–2170. 23937440 10.1111/jeb.12212

[brv70062-bib-0131] Uller, T. & Pen, I. (2011). A theoretical model of the evolution of maternal effects under parent–offspring conflict. Evolution 65, 2075–2084. 21729061 10.1111/j.1558-5646.2011.01282.x

[brv70062-bib-0132] Van Cann, J. , Koskela, E. , Mappes, T. , Sims, A. & Watts, P. C. (2019). Intergenerational fitness effects of the early life environment in a wild rodent. Journal of Animal Ecology 88, 1355–1365. 31162628 10.1111/1365-2656.13039

[brv70062-bib-0133] van den Heuvel, J. , English, S. & Uller, T. (2016). Disposable soma theory and the evolution of maternal effects on ageing. PLoS One 11, e0145544. 26752635 10.1371/journal.pone.0145544 PMC4709080

[brv70062-bib-0134] Waddington, C. H. (1942). Canalization of development and the inheritance of acquired characters. Nature 150, 563–565. 10.1038/1831654a0 13666847

[brv70062-bib-0135] Wei, H. , He, X. J. , Liao, C. H. , Wu, X. B. , Jiang, W. J. , Zhang, B. , Zhou, L. B. , Zhang, L. Z. , Barron, A. B. & Zeng, Z. J. (2019). A maternal effect on queen production in honeybees. Current Biology 29, 2208–2213. 31231048 10.1016/j.cub.2019.05.059

[brv70062-bib-0136] Wells, J. C. K. (2007). The thrifty phenotype as an adaptive maternal effect. Biological Reviews 82, 143–172. 17313527 10.1111/j.1469-185X.2006.00007.x

[brv70062-bib-0137] West‐Eberhard, M. J. (2003). Developmental Plasticity and Evolution. Oxford University Press, Oxford.

[brv70062-bib-0138] West‐Eberhard, M. J. (2005). Phenotypic accommodation: adaptive innovation due to developmental plasticity. Journal of Experimental Zoology Part B: Molecular and Developmental Evolution 304, 610–618. 16161068 10.1002/jez.b.21071

[brv70062-bib-0139] Wilson, A. J. & Nussey, D. H. (2010). What is individual quality? An evolutionary perspective. Trends in Ecology & Evolution 25, 207–214. 19897275 10.1016/j.tree.2009.10.002

[brv70062-bib-0140] Wilson, A. J. , Reale, D. , Clements, M. N. , Morrissey, M. M. , Postma, E. , Walling, C. A. , Kruuk, L. E. & Nussey, D. H. (2010). An ecologist's guide to the animal model. Journal of Animal Ecology 79, 13–26. 20409158 10.1111/j.1365-2656.2009.01639.x

[brv70062-bib-0141] Wolf, J. B. , Brodie, E. D. III , Cheverud, J. M. , Moore, A. J. & Wade, M. J. (1998). Evolutionary consequences of indirect genetic effects. Trends in Ecology & Evolution 13, 64–69. 21238202 10.1016/s0169-5347(97)01233-0

[brv70062-bib-0142] Wolf, J. B. & Wade, M. J. (2009). What are maternal effects (and what are they not)? Philosophical Transactions of the Royal Society B: Biological Sciences 364, 1107–1115. 10.1098/rstb.2008.0238 PMC2666680 19324615

[brv70062-bib-0143] Zhou, L. & Declerck, S. A. J. (2020). Maternal effects in zooplankton consumers are not only mediated by direct but also by indirect effects of phosphorus limitation. Oikos 129, 766–774.

